# Efficacy of Durvalumab Consolidation Therapy After Sequential Chemoradiotherapy in Patients with Unresectable Stage III Non-Small Cell Lung Cancer—Experience from the Daily Hospital of Clinic for Pulmonology, University Clinical Center of Serbia

**DOI:** 10.3390/biomedicines13040892

**Published:** 2025-04-07

**Authors:** Vesna Ćeriman Krstić, Natalija Samardžić, Spasoje Popević, Ruža Stević, Branislav Ilić, Milija Gajić, Nikola Čolić, Katarina Lukić, Brankica Milošević Maračić, Bojana Poparić Banđur, Biljana Šeha, Damir Radončić, Jelena Milin Lazović

**Affiliations:** 1 Faculty of Medicine, University of Belgrade, 11000 Belgrade, Serbia; 2 Clinic for Pulmonology, University Clinical Center of Serbia, 11000 Belgrade, Serbia; 3 Center for Radiology, University Clinical Center of Serbia, 11000 Belgrade, Serbia; 4 Institute of Oncology and Radiology Serbia, 11000 Belgrade, Serbia; 5 Clinic for Neurosurgery, University Clinical Center of Serbia, 11000 Belgrade, Serbia; 6 Institute of Medical Statistics, Faculty of Medicine, University of Belgrade, 11000 Belgrade, Serbia

**Keywords:** stage III NSCLC, durvalumab, radiotherapy, real-world data, PFS, OS

## Abstract

**Background/Objectives:** Patients with stage III non-small cell lung cancer represent a very heterogeneous group of patients. In the past, the standard of care for patients with inoperable stage III non-small cell lung cancer was concurrent or sequential radical radiotherapy and chemotherapy. But the progression-free survival was 8 months, and the 5-year overall survival rate was less than 20%. After the results of the PACIFIC study, the standard of care for this group of patients is chemoradiotherapy with durvalumab as consolidation therapy. The aim of our study was to evaluate the efficacy of consolidation durvalumab in a real-world setting after sequential CRT. **Methods:** We included 24 patients with unresectable stage III non-small cell lung cancer who did not progress after sequential chemoradiotherapy and who received durvalumab consolidation. **Results:** Median progression-free survival was 16 months, 95% CI (0.5–31.5), and median overall survival was 20 months, 95% CI (13.4–26.6 months). The twelve-month progression-free survival and overall survival rate were 55.1% and 68%, respectively, and the 18-month progression-free survival and overall survival rates were 44.1% and 56.5%, respectively. **Conclusions:** Durvalumab introduced a new era in the treatment of patients with unresectable stage III non-small cell lung cancer with a significantly prolonged 5-year overall survival rate. Our study is one of the few that investigated the efficacy of durvalumab in a real-world setting after sequential CRT. Our results showed that durvalumab is effective in patients who were treated with sequential CRT. However, the time between radiotherapy termination and the start of durvalumab should be shorter.

## 1. Introduction

Patients with stage III non-small cell lung cancer (NSCLC) represent a very heterogeneous group of patients that could be very difficult to treat. They could be candidates for surgery, but they could also have inoperable tumors, and in that case, the standard of care in the past was concurrent or sequential radical radiotherapy and chemotherapy [1]. For this group of patients the progression-free survival (PFS) was 8 months, and the 5-year overall survival (OS) rate was less than 20% [1].

Curran et al. [2] conducted a randomized trial to investigate which strategy could be optimal in patients with inoperable stage III NSCLC: sequential or concurrent delivery of radiotherapy. They included 610 patients in their analysis. It was shown that patients treated with concurrent chemoradiotherapy (CRT) had statistically better OS: 14.6 months for patients treated with sequential CRT compared to 17 and 15.6 months for patients treated with concurrent CRT [2]. Five-year OS rates were 10% for patients treated with sequential CRT compared to 16% and 13% for the patients treated with concurrent CRT [2].

In the past, several trials evaluated the role of chemotherapy as consolidation, but the results did not show significant benefit [3,4,5,6]. Similar results were obtained from the meta-analysis conducted by Tsujino et al. [7].

Therefore, there was a need for effective consolidation therapy. Nowadays, after the results of the PACIFIC trial [8], the standard of care for patients with stage III inoperable NSCLC without driver mutations is concurrent CRT with durvalumab as consolidation therapy. Also, the results of PACIFIC 6 showed comparable efficacy of durvalumab when sequential radiotherapy was applied [9].

The aim of our study was to evaluate the efficacy of consolidation durvalumab in a real-world setting after sequential CRT.

## 2. Materials and Methods

### 2.1. Patients and Data Collection

We enrolled 24 patients treated with consolidation durvalumab who had unresectable stage III NSCLC and who did not progress after sequential radical CRT. All included patients had a programmed cell death-ligand 1 (PD-L1) expression ≥ 1%, and they were all treated at Daily Hospital of Clinic for Pulmonology, University Clinical Center of Serbia. All patients were treated with durvalumab. All patients met the following inclusion criteria: histopathologically confirmed NSCLC; without epidermal growth factor receptor (EGFR) and anaplastic lymphoma kinase (ALK) mutations; PD-L1 expression ≥ 1%; unresectable stage III of disease (AJCC 8th edition); none of them showing progression of disease on previously applied sequential radical CRT; measurable disease at baseline; ECOG PS 0 or 1. Exclusion criteria: histopathologically confirmed other subtypes of lung carcinoma; early or metastatic stage of disease; PD-L1 expression < 1%; progression of disease on previously applied sequential radical CRT; patients with unresectable stage III who did not received a radical dose of radiotherapy; ECOG PS ≥ 2. All patients underwent a computed tomography of the chest and upper abdomen and head or magnetic resonance of the head at baseline to determine the stage of disease. The data about sex, age, smoking status [i.e., non-smokers, ex-smokers (patients who stopped smoking one year before treatment), smokers], stage of the disease, response to the therapy (ORR), PFS, and OS were collected retrospectively. The response to therapy included complete response (CR), partial response (PR), stable disease (SD)n and progression of disease (PD). The PFS was calculated from the last day of radiotherapy until disease progression or death, and the OS was calculated from the last day of radiotherapy until death from any cause. The non-interventional, observational study was conducted in accordance with the Declaration of Helsinki and it was approved by the local Institutional Board.

### 2.2. Statistical Analysis

The results are presented as count (%) or means ± standard deviation, depending on data type. A Kaplan–Meier with Log-ranks test was used to assess survival and group differences regarding survival. Survival is presented using median (95% CI) or percentage of participant without event of interest in specific time period. All *p* values less than 0.05 have been considered significant. All data were analyzed using SPSS 29.0 (IBM Corp. Released 2023, IBM SPSS Statistics for Windows, Version 20.0. IBM Corp., Armonk, NY, USA).

## 3. Results

Twenty-four patients with unresectable stage III NSCLC who had no progression on previous sequential CRT and who had a PD-L1 expression ≥ 1%, negative EGFR and ALK mutations were included in the analysis. The median follow-up time was 13 months (minimum for 4 months and the maximum was 27 months). All patients received durvalumab at the dose of 1500 mg every four weeks, except one, who received durvalumab every two weeks at the dose of 10 mg/kg. Patients were previously treated with platinum doublet. Main baseline demographic characteristics of included patients have been presented in Table 1. The majority of patients received platinum in combination with paclitaxel and gemcitabine, 10 and 11, respectively. Eleven patients received six cycles of chemotherapy before radiotherapy, and ten of them received four cycles. Median number of durvalumab application was 7.7. Radical sequential radiotherapy was applied in total dose of 54–66 Gy, 2 fractions by day. The majority of patients received a total dose of 60 Gy/30 fractions.

The median time from termination of radiotherapy until durvalumab initiation was 98.9 days (ranging from 30 to 231 days). At the time of the data cut-off, one half of the patients did not have disease progression, and 66.7% were still alive.

**Figure 1:**
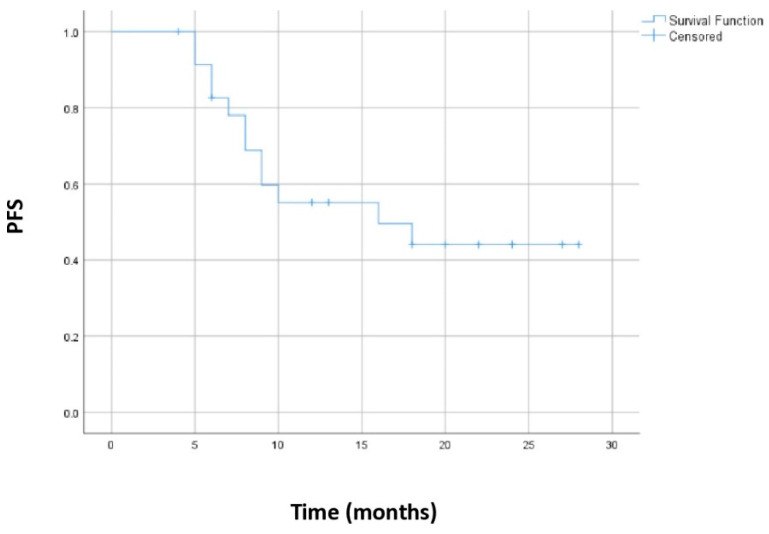
Kaplan–Meier curve for progression-free survival for the whole group of patients.

Median progression-free survival was 16 months, 95% CI (0.5–31.5). Figure 1 shows the Kaplan–Meier curve for the PFS for the whole group of patients.

**Figure 2:**
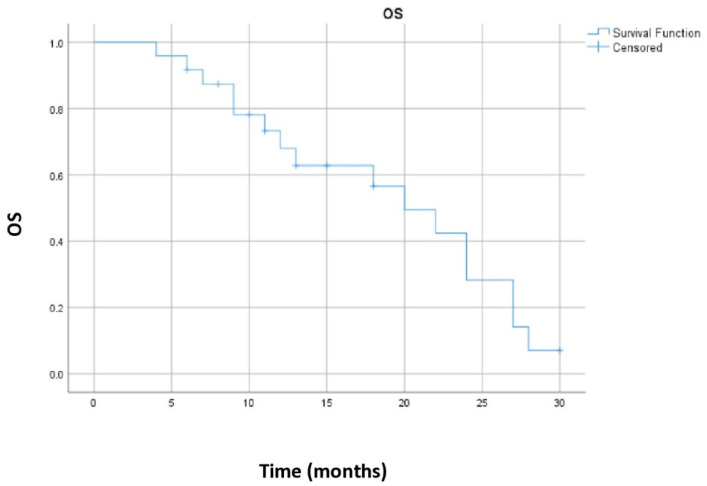
Kaplan–Meier curve for the overall survival for the whole group of patients.

Median overall survival was 20 months, 95% CI (13.4–26.6 months). Figure 2 shows the Kaplan–Meier curve for the OS for the whole group of patients.

**Figure 3:**
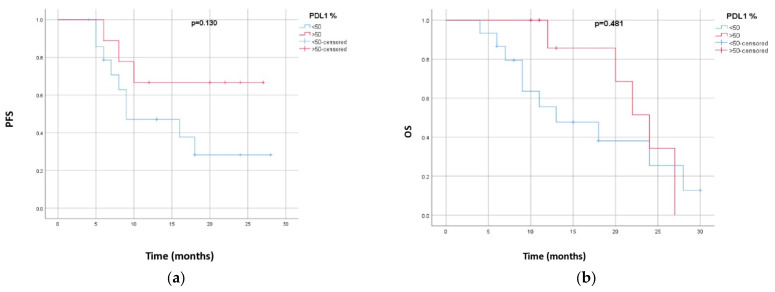
(**a**) Progression-free survival depending on PD-L1 expression. (**b**) Overall survival depending on PD-L1 expression.

There were no significant differences in the PFS and OS by sex, smoking status, or PD-L1 expression. Although patients with a PD-L1 expression ≥ 50% had numerically better PFS and OS rates (the difference was more than 5 months), the differences were not significant. Table 2 shows the results for PFS and OS depending on sex, smoking status, and PD-L1 expression. Figure 3a,b show PFS and OS depending on the PD-L1 expression.

Six-month PFS and OS rates were 91.3% and 91.7%, respectively. Further, 12-month PFS and OS rates were 55.1% and 68%, respectively, and 18-month PFS and OS rates were 44.1% and 56.5%, respectively.

Half of the patients had disease progression; 20.8% had local progression, 29.2% had systemic progression and 16.7% developed brain metastases.

No grade ≥ 3 toxicity was recorded.

## 4. Discussion

We evaluated the efficacy of consolidation durvalumab in 24 patients with unresectable stage III NSCLC who did not have disease progression after sequential CRT in a real-world setting.

The PACIFIC trial included 709 patients who received consolidation durvalumab or placebo after concurrent CRT who had no disease progression [8]. The median PFS was 16.8 months with durvalumab compared to 5.6 months with a placebo [8]. The median PFS in our study was similar. The 12-month PFS rate was 55.9% (similar to our results) versus 35.3%, and the 18-month PFS rate was 44.2% compared to 27%. The median time to death or appearance of distant metastases was 23.2 months with durvalumab compared to 14.6 months with a placebo [8]. Patients treated with durvalumab had a lower incidence of brain metastases compared to patients treated with a placebo, 5.5% versus 11% [8]. In our study, 16.7% of patients developed brain metastases; it might be due to the prolonged time until the initiation of durvalumab. The 24-month OS rate in the PACIFIC trial was 66.3% compared to 55.6% [10].

The subgroup analysis showed that patients treated with durvalumab had a longer PFS regardless of the PD-L1 expression, and regarding OS, the benefit was shown in patients with a PD-L1 expression ≥ 1%, but not in a group of patients with a PD-L1 expression less than 1% [11].

The 12-, 24-, and 36-month OS rates with durvalumab were 83.1% versus 74.6% with a placebo, 66.3% compared to 55.3% with a placebo, and 57% versus 43.5% with a placebo, respectively [12]. In our study, the 12-month OS rate was 68%.

Further, the 5-year OS rate for patients treated with durvalumab was 42.9% compared to 33.4% for the placebo group, and the 5-year PFS rate was 33.1% versus 19% for the placebo group [13]. The median OS was 47.5 months with durvalumab compared to 29.1 months with the placebo [13]. The incidence of new lesions development was lower with durvalumab (24.2%) compared to the placebo (33.3%) [13]. An updated analysis showed that after more than 5 years of follow up, 6.5% of patients treated with durvalumab developed brain metastases compared to 11.8% in a group of patients treated with a placebo [13]. The results are similar to those previously reported. Both univariate and multivariable analyses confirmed that younger age, ECOG PS 0, non-squamous histology, and Asian race were favorable prognostic factors for OS [13], and Asian race and non-squamous histology were favorable prognostic factors for PFS [13]. A multivariable analysis did not confirm that cisplatin use during prior CRT and objective tumor response during prior CRT were favorable prognostic factors for OS [13].

The PACIFIC 6 trial evaluated the efficacy of durvalumab after sequential CRT, and it was shown that the results were comparable with the results shown for patients treated with concurrent CRT [9]. There were 117 patients enrolled, and among them, 3 patients had ECOG PS 2, but that did not affect the results. This study also included patients with a PD-L1 expression < 1%, approximately 30%, and for 40% of patients, the results for PD-L1 expression were missing. The median PFS was 10.9 months, and 12-month PFS and OS rates were 49.6% and 84.1%, respectively [9]. In our study, mPFS was 16 months, and the 12-month PFS and OS rates were 55.1% and 68%, respectively. However, in the PACIFIC 6 trial, there were 53.8% of included patients with adenocarcinoma histology, and in our study, only one-third of patients had adenocarcinoma histology.

The PACIFIC-R study included 1399 patients with stage III NSCLC who received durvalumab after CRT [14]. In contrast to the PACIFIC trial, this study also included patients with poor ECOG PS and patients treated with sequential CRT [14]. The majority of patients, 64%, had non-squamous histology [14], while in our study, the majority of patients had squamous histology. The median time from termination of RT until initiation of durvalumab was 56 days (ranging from 35 to 981 days). Only 30.1% of patients started durvalumab within 42 days after the termination of RT, and in 14.4%, the treatment with durvalumab was started after more than 3 months [14]. The median PFS was 21.7 months. The 12- and 24-month PFS rates were 62.2% and 48.2%, respectively [14]. The PFS was numerically longer in patients who were treated with concurrent CRT, in patients with a PD-L1 ≥ 1%, in patients with stage IIIa, and in patients with non-squamous NSCLC, and also in patients who received cisplatin and in patients who started durvalumab within 42 days after completion of radiotherapy [14]. The median OS was not reached, and the 24-month OS rate was 71.2% [14].

The Pacific-KR study [15] involved 157 patients who were treated with durvalumab after CRT. The PFS was 25.9 months, and 1-, 2-, and 3-year PFS rates were 59.4%, 51.8%, and 43.5%, respectively [15]. The ORR was 51%, and the OS was not reached, and 1-, 2- and 3-year OS rates were 87.8%, 71%, and 69.2%, respectively [15]. Also, it was shown that patients with a high PD-L1 had a longer PFS [15]. The majority of patients had locoregional progression (56.2%), and regarding distant sites of progression, the predominant sites were brain and bones, with 13.7% each [15].

Huang et al. [16] conducted a retrospective study and enrolled 84 patients with stage III who received concurrent chemoradiotherapy, and 39 of them were treated with durvalumab consolidation. The majority of patients had non-squamous histology and received durvalumab within 42 days after radiotherapy termination [16]. The median PFS was 17.5 months and median OS was not reached in a group of patients treated with durvalumab [16]. The majority of patients had systemic progression in both groups of patients [16].

In a study conducted by Chu et al. [17], the majority of patients also had non-squamous histology. The median time from concurrent chemoradiotherapy completion until durvalumab initiation was 2.8 months [17]. The 1-year PFS rate was 56.4%, and the median PFS was not reached [17].

Preti et al. [18] retrospectively investigated the efficacy of durvalumab consolidation in 118 patients, but this study also included patients with stage IV or an unknown stage of disease and patients with a PD-L1 < 1%, and like in previously mentioned studies, the majority of patients had non-squamous histology. The median time from completion of chemoradiotherapy until durvalumab initiation was 40.5 days (ranging from 7 to 238 days) [18]. The median PFS and OS were not reached, but were estimated to be >20 and >32 months, respectively [18].

Taugner et al. [19] included 144 patients in the analysis, and among them, 22 patients received durvalumab consolidation therapy. The study was partly retrospective and partly prospective (patients were treated with durvalumab after the EMA approval), and 12- and 18-month OS rates were 100% and 91.6%, respectively, in patients treated with durvalumab consolidation [19]. The median PFS was not reached, and 12- and 18-month PFS rates were 60% and 43.8%, respectively, for patients who received durvalumab consolidation therapy [19].

In another prospective study, conducted by Taugner et al. [20], 26 patients were included in the analysis. The predominant histology was also non-squamous. The median PFS was not reached, and 6- and 12-month OS rates were 100% and 100%, and 6- and 12-month PFS rates were 82% and 62%, respectively [20]. Durvalumab was interrupted in 27% of patients due to disease progression, and there were no cases of hyperprogression [20].

Faehling et al. [21] retrospectively evaluated the efficacy of durvalumab in 126 patients who received at least one cycle of durvalumab. The analysis also included patients with oligometastatic disease, 5.6% of them, and patients with a negative PD-L1 expression [21]. More than half of patients (51.6%) had adenocarcinoma histology and 42.6% had squamous cell carcinoma, [21]. The PFS was 20.1 months, and the OS was not reached [21]. The 12- and 18-month PFS rates were 56% and 52.8%, respectively, and the 12- and 18-month OS rates were 78.6% and 66%, respectively [21]. The median time to death or the development of distant lesions was not reached [21]. The incidence of brain metastases was 6.3% [21].

Jung et al. [22] conducted a retrospective non-interventional observational study that evaluated the efficacy of consolidation durvalumab in 21 patients after concurrent CRT. Also, 40 patients were observed after concurrent CRT, and they did not receive durvalumab consolidation [22]. The majority of patients (60.7%) had squamous cell lung cancer, 44.3% had stage IIIc, and 14.3% had EGFR-positive NSCLC [22]. The PFS was not reached in the durvalumab group compared to 9.6 months for the observational group [22]. A comparative view of different studies that evaluated the efficacy of consolidation durvalumab has been presented in Table 3.

According to our knowledge, our study is the only one besides PACIFIC 6 that investigated the efficacy of durvalumab in a real-world setting after sequential CRT. The PFS in our study was comparable with the results of PACIFIC 6 and other studies, but the OS was lower compared to other studies. In our study, the majority of patients had squamous histology, and in other studies, the predominant histology was non-squamous. Also, when we analyzed the outcomes by subgroup, the results showed that the differences were not significant. In a subgroup of patients with a PD-L1 ≥ 50%, the differences in PFS and OS rates were numerically better (more than 5 months) than in a subgroup of patients with a PD-L1 < 50%, but without statistical significance, possibly due to the small number of patients.

The main limitation of our study is the small number of patients. We included 24 patients in our study. Further, the median time from the termination of radiotherapy until the initiation of durvalumab was longer in our study compared to the registration study (this is due to technical limitations—durvalumab is not covered by our insurance fund), which could explain the lower 12-month OS rate in our study and the increased percentage of patients who developed brain metastases. Also, in the majority of the presented studies, patients with non-squamous histology were predominant, and in our study, they represented approximately half of the included patients.

## 5. Conclusions

Patients with stage III NSCLC are a very heterogeneous group, which is very hard to treat. Durvalumab introduced a new era in the treatment of patients with unresectable stage III non-small cell lung cancer with a significantly prolonged 5-year overall survival rate. Our study is one of the few that investigated the efficacy of durvalumab in a real-world setting after sequential CRT. Our results showed that durvalumab is effective in patients who were treated with sequential CRT. However, the time between radiotherapy termination and the start of durvalumab should be shorter.

## Figures and Tables

**Table 1:** Main baseline demographic characteristics of included patients.

Demographic Characteristics	All Patients (N = 24)
Age, mean ± SD	66.3 ± 4.6
Sex	
Female sex, n (%)	9 (37.5)
Male sex, n (%)	15 (62.5)
Smoking	
Former smokers, n (%)	13 (54.2)
Current smokers, n (%)	11 (45.8)
Tumor histologic type	
Adeno, n (%)	8 (33.3)
Squamous, n (%)	12 (50.0)
Large cell, n (%)	1 (4.2)
NOS, n (%)	3 (12.5)
Stage (AJCC 8th edition)	
IIIa, n (%)	11 (45.8)
IIIb, n (%)	11 (45.8)
IIIc, n (%)	2 (8.3)
PD-L1 expression	
≥50%, n (%)	9 (37.5)
1–49%, n (%)	15 (62.5)
Previous chemotherapy	
Ethoposide, Platinum, n (%)	2 (8.3)
Vinorelbine, Platinum, n (%)	1 (4.2)
Gemcytabine, Platinum, n (%)	11 (45.8)
Paclitaxel, Platinum, n (%)	10 (41.7)
ECOG PS	
ECOG PS 0, n (%)	2 (8.0)
ECOG PS 1, n (%)	22 (92)

**Table 2:** PFS and OS depending on sex, smoking status, and PD-L1 expression.

	PFS	*p* Value	OS	*p* Value
Sex		*p* = 0.617		*p* = 0.675
Male	18.5 (13.4–23.6)		19.0 (14.6–23.4)	
Female	15.2 (8.7–21.8)		18.2 (11.6–24.9)	
Smoking status		*p* = 0.823		*p* = 0.607
Former smokers	16.5 (10.7–22.3)		18.2 (12.8–23.6)	
Current smokers	18.2 (12.7–23.7)		19.1 (14.2–24.1)	
PD-L1 expression		*p* = 0.13		*p* = 0.481
≥50%	20.7 (14.8–26.5)		22.3 (18.2–26.4)	
1–49%	14.9 (9.8–19.9)		16.6 (11.6–21.7)	

*p* value log-rank test.

**Table 3:** A comparative view of different studies that evaluated the efficacy of consolidation durvalumab.

Study	No of Patients	Concurrent/ Sequential RT	PFS (Months)	OS (Months)	12-Month PFS Rate	12-Month OS Rate
PACIFIC [8]	709	Concurrent	16.8	47.5	55.9%	83.1%
PACIFIC-R [14]	1399	Both	21.7	Not reached	62.2%	/
PACIFIC-KR [15]	157	Both	25.9	Not reached	59.4%	87.8%
PACIFIC 6 [9]	117	Sequential	10.9	/	49.6%	84.1%
Huang et al. [16]	39	Concurrent	17.5	Not reached	/	/
Chu et al. [17]	31	Concurrent	Not reached	/	56.4%	/
Preti et al. [18]	118	Concurrent	Not reached >20 months	Not reached >32 months	/	/
Taugner et al. [19]	26	Both	Not reached	/	62%	100%
Faehlig et al. [21]	126	Concurrent	20.1	Not reached	56%	78.6%
Ceriman Krstic and Samardzic et al.	24	Sequential	16	20	55.1%	68%

## Data Availability

Data is contained within the article.
